# Supplementary material for: Hard rock landforms generate 130 km ice shelf channels through water focusing in basal corrugations
Source: Nat Commun. 2018 Nov 1;9:4576. doi: 10.1038/s41467-018-06679-z (PMC6212400; doi:10.1038/s41467-018-06679-z)
Supplement: Supplementary file 1 — Supplementary Information [file 41467_2018_6679_MOESM1_ESM.pdf]

Hard rock landforms generate 130 km ice shelf channels through water focusing in basal corrugations

Jeofry et al.

Supplementary Information

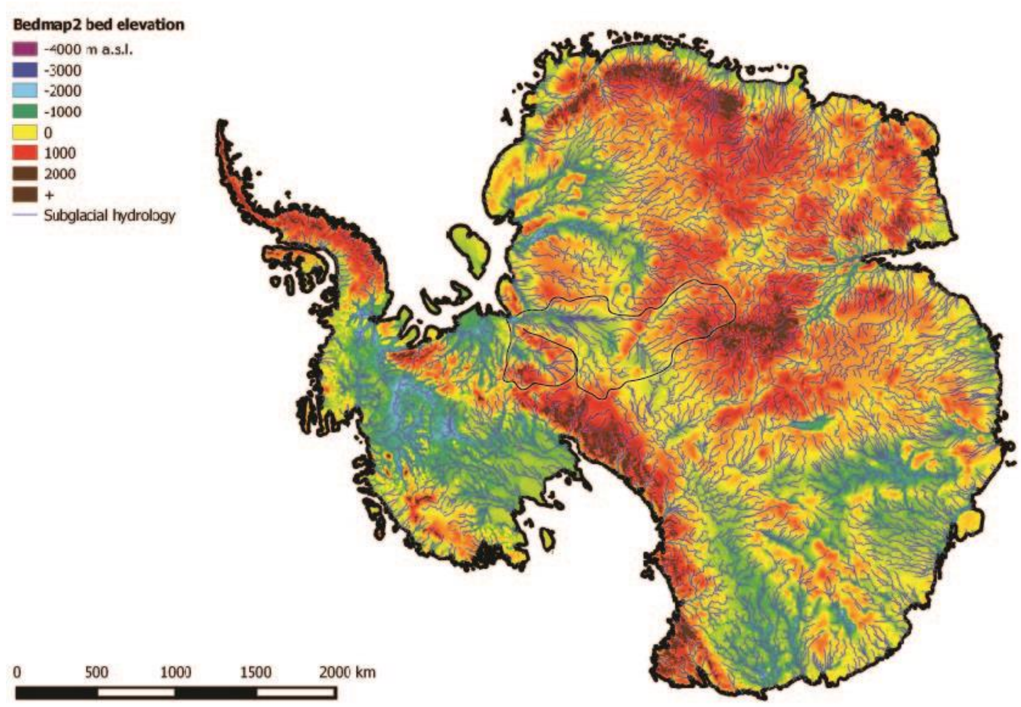

Supplementary Figure 1. Subglacial hydrological pathways for the whole of Antarctica, using Bedmap2 ice-surface and bed-elevation data[1], revealing the approximate catchments (black lines) of the Foundation Ice Stream in East and West Antarctica. The maximum possible size of the sub-glacial drainage systems are shown, assuming the bed is at the pressure melting point and thawed at all points.

(a)

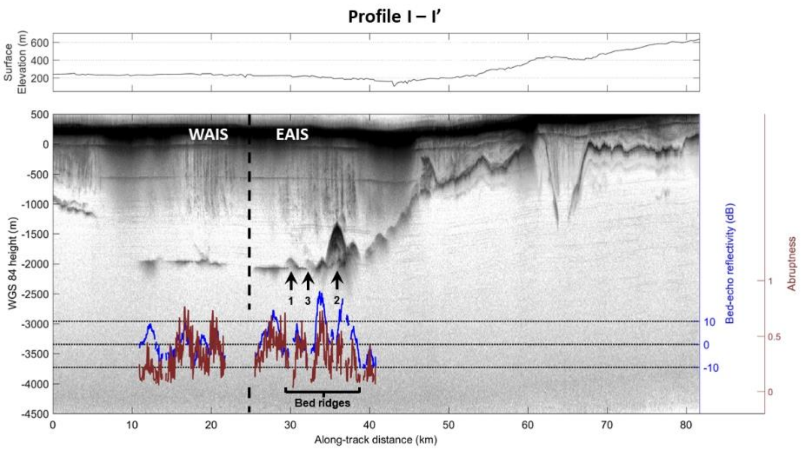

(b)

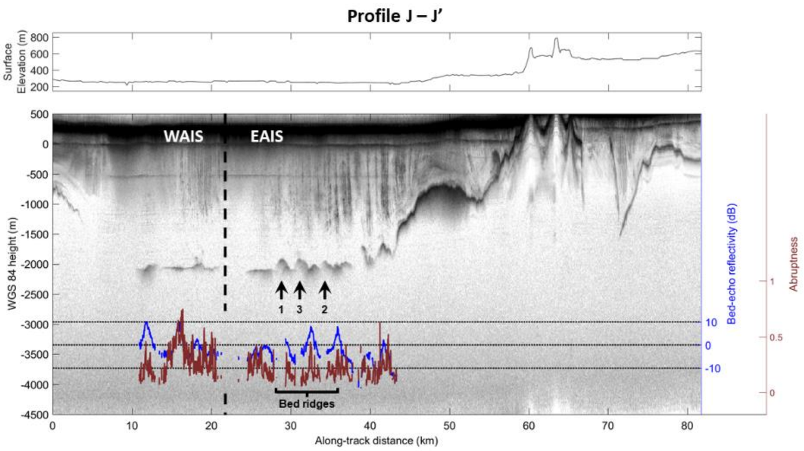

(c)

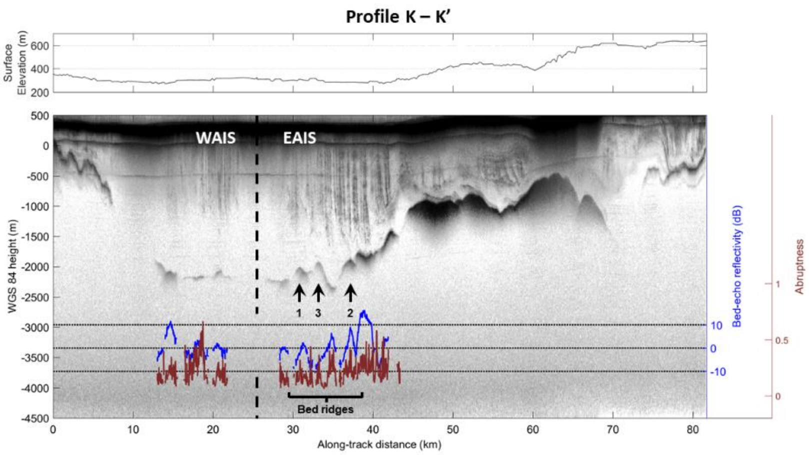

Supplementary Figure 2. Radar transects for (a) I-I’, (b) J-J’ and (c) K-K’, representing expanded versions of those in Figure 2 (main paper) revealing the broader topographic context of Foundation Ice Stream. Locations are provided in Figure 1a.

Supplementary Note 1. RES reflectivity analysis

Radar profiles used in the basal reflectivity analysis (I-I', J-J' and K-K') are from the same flight of the 2012 field season (Oct 28th) using the MCoRDs radar system onboard a DC8 aircraft (see Ref [6] for more details). The technique to extract basal power from the radar echo-strength profiles (L1B data)[7], aggregates power over bed-echo fading (i.e. performs a depth-range integral), the rationale being that this acts to reduce the effects of roughness-induced scattering loss[8]. A signal-to-noise threshold was imposed, such that the peak basal power was > 5 dB above the noise floor. The abruptness of the bed-echo was then calculated from the ratio of peak to aggregated power[7, 8]. Prior to the reflectivity analysis, the basal power was geometrically corrected using L2 ice thickness data. To calculate (relative) basal reflectivity from basal power it is necessary to account for attenuation losses, which are an Arrhenius function of temperature and chemistry[9]. Often when considering a region where ice thickness (radar path length) is relatively constant, it is sufficient to use a single attenuation correction (depth-averaged attenuation rate). However, the main trunk of the FIS represents an unusual scenario where East and West Antarctic ice intersect, and it is therefore reasonable to assume that the attenuation properties are different in each ice mass. Supporting evidence for this anticipated step-change in attenuation is that basal power values from East Antarctica ice are systematically higher than West Antarctic ice in the main trunk of the FIS by ~6 dB). To accommodate for this unusual scenario, we masked the RES power data into East and West regions within the main trunk (corresponding to data either side of the coverage gaps along profiles I-I', J-J' and K-K'). We then considered attenuation corrections of the form $\langle N\rangle-\Delta/2$ dB/km and $\langle N\rangle+\Delta/2$ dB/km in East and West sections of the main trunk where $\langle N\rangle$ is the mean (depth-averaged) attenuation rate and Δ is the attenuation difference between West and East sides of the main trunk. Posing this as an optimization problem, we then solved for the values of $\langle N\rangle$ and Δ that minimized the overall standard deviation of basal reflectivity, obtaining values of $\langle N\rangle=10.0$ dB/km and $\Delta=1.0$ dB/km. These inferred attenuation values are toward the lower end of the range predicted for Antarctic ice streams[10] and are consistent with the fact that the FIS is one of the furthest inland and coldest ice streams in Antarctica. They also implicate that the East Antarctic ice is either colder, and/or has significantly less ionic impurities than West Antarctic ice. The combined basal reflectivity distribution using the separate attenuation corrections for East and West sections of the main trunk is shown in Supplementary Figure 3a. The reflectivity values are normalized about zero, the standard deviation is 6.1 dB, and approximately 98 % of the data is contained within a 30 dB range. A frequency distribution for the bed-echo abruptness is shown in Supplementary Figure 3b. The values range from ~ 0.05 to 0.80 with lower values associated with diffuse scattering and higher values associated with specular reflections.

We did not interpret basal reflectivity values across the grounding line (transect L-L'), due to the rapidly varying attenuation structure in these regions[11]. However, the abruptness parameter is near attenuation independent and provides a robust means to identify the basal transition.

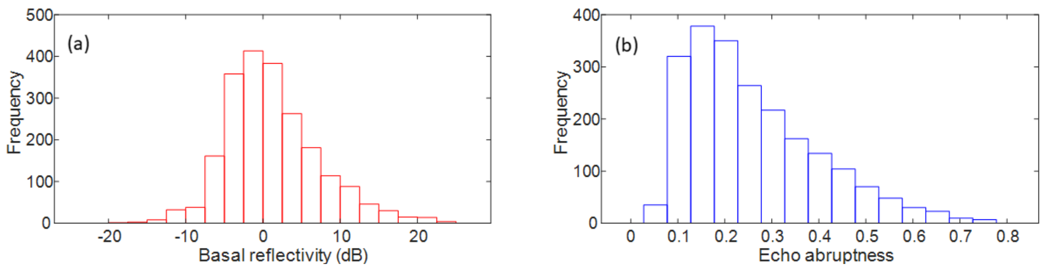

Supplementary Figure 3. Frequency distributions for (a) basal reflectivity and (b) echo abruptness, in the main trunk of the FIS (East and West regions of transects I-I', J-J', K-K'; Figure 2 of main paper).

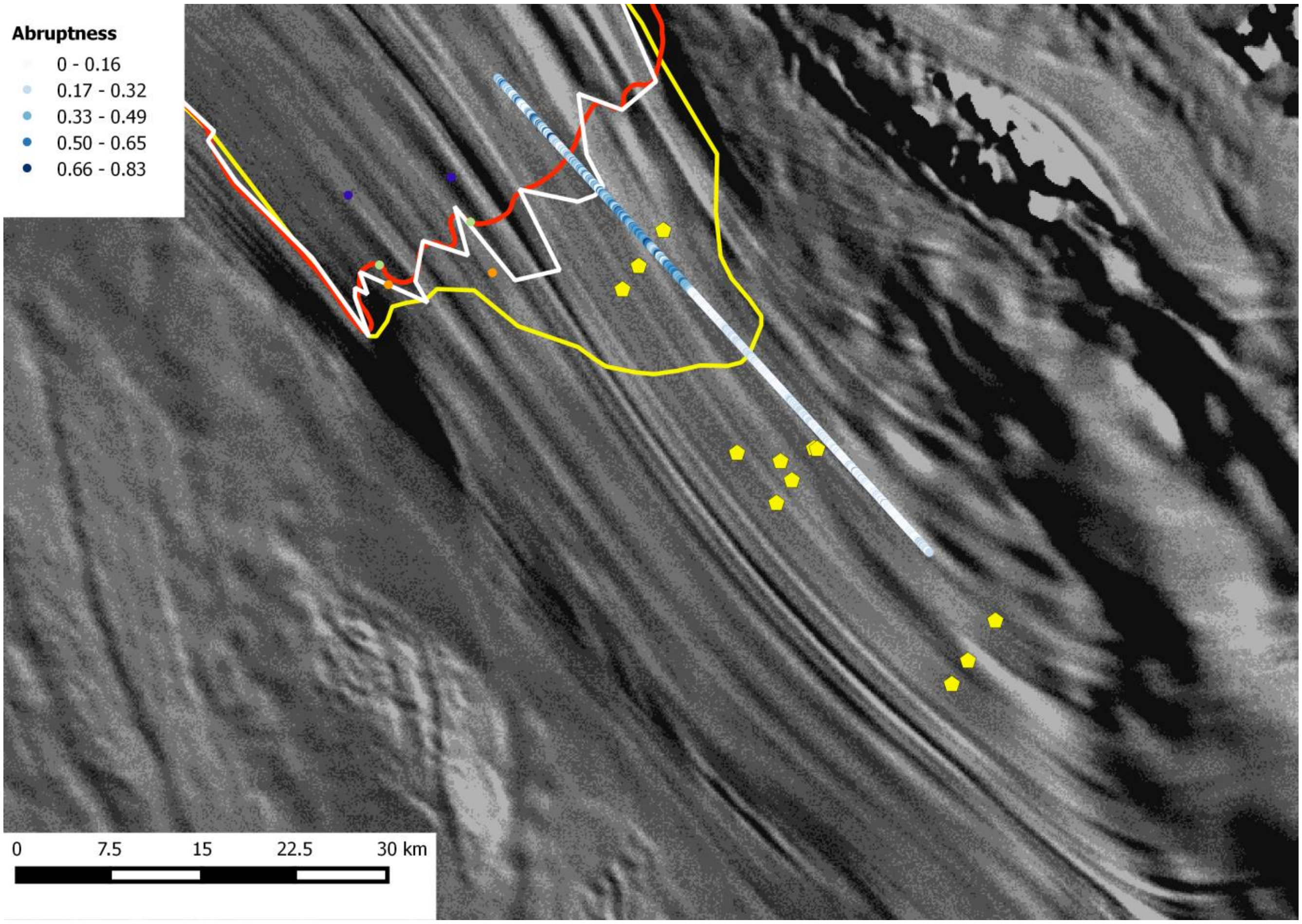

Supplementary Figure 4. Peaks of the subglacial bedforms (yellow pentagons), derived from radar data (Figure 2 of the main paper), superimposed over MODIS ice-surface imagery revealing an alignment between flowstripes and bedforms over grounded ice. Grounding points from Ice, Cloud and land Elevation Satellite (ICESat) laser altimetry are as follows: blue – hydrostatic point; orange – ice flexure landward limit; green – break in slope[2]. Grounding lines from the Antarctic Surface Accumulation and Ice Discharge (ASAID) are in red[3]. Grounding lines from the Differential Satellite Synthetic Aperture Radar Interferometry (DInSAR) are in yellow[4]. Grounding lines from the Mosaic of Antarctica (MOA) are in white[5]. Also shown is a depiction of the basal reflectivity abruptness (white-blue line; see N-N' in Figure 2 of main paper), showing a step-change in abruptness and revealing that the grounding zone initiates upstream two landform measurements and the 800 m basal peak interpreted as the start of a U-channel in Line I-I' (Figure 2 of main paper).

(a)

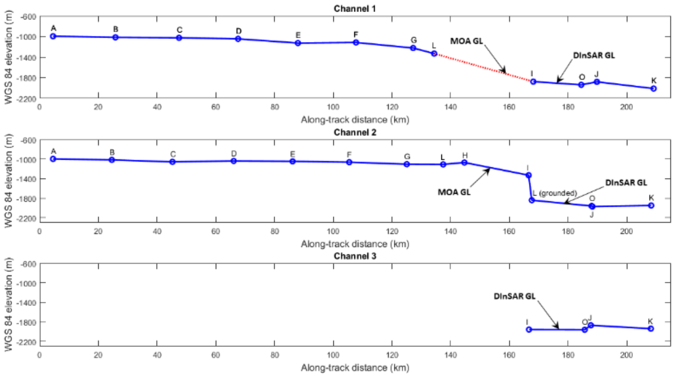

Elevation

(b)

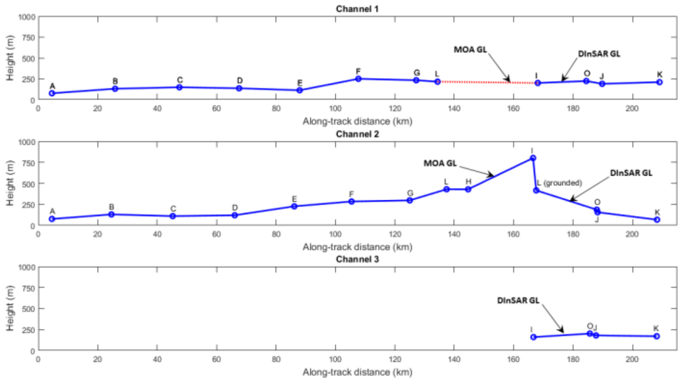

Height

(c)

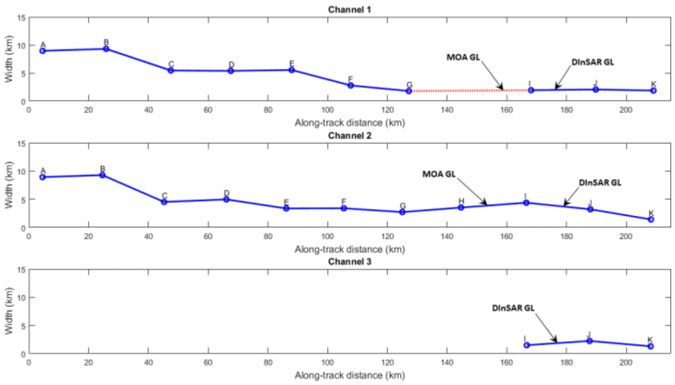

Width

Supplementary Figure 5. Dimensions of measured bedforms and U-channels. (a) Elevation, (b) height and (c) widths, of three bedforms and U-channels, measured at each transect and followed between transects using flowstripes. Intersection points with radargrams are labelled as in Figure 1. Locations of the three bedforms/U-channels are provided in Figure 2 of main paper. The locations of the bed features are given in Supplementary Figure 6. Also shown are the locations of DInSAR (flexure) and MOA surface slopes) grounding lines.

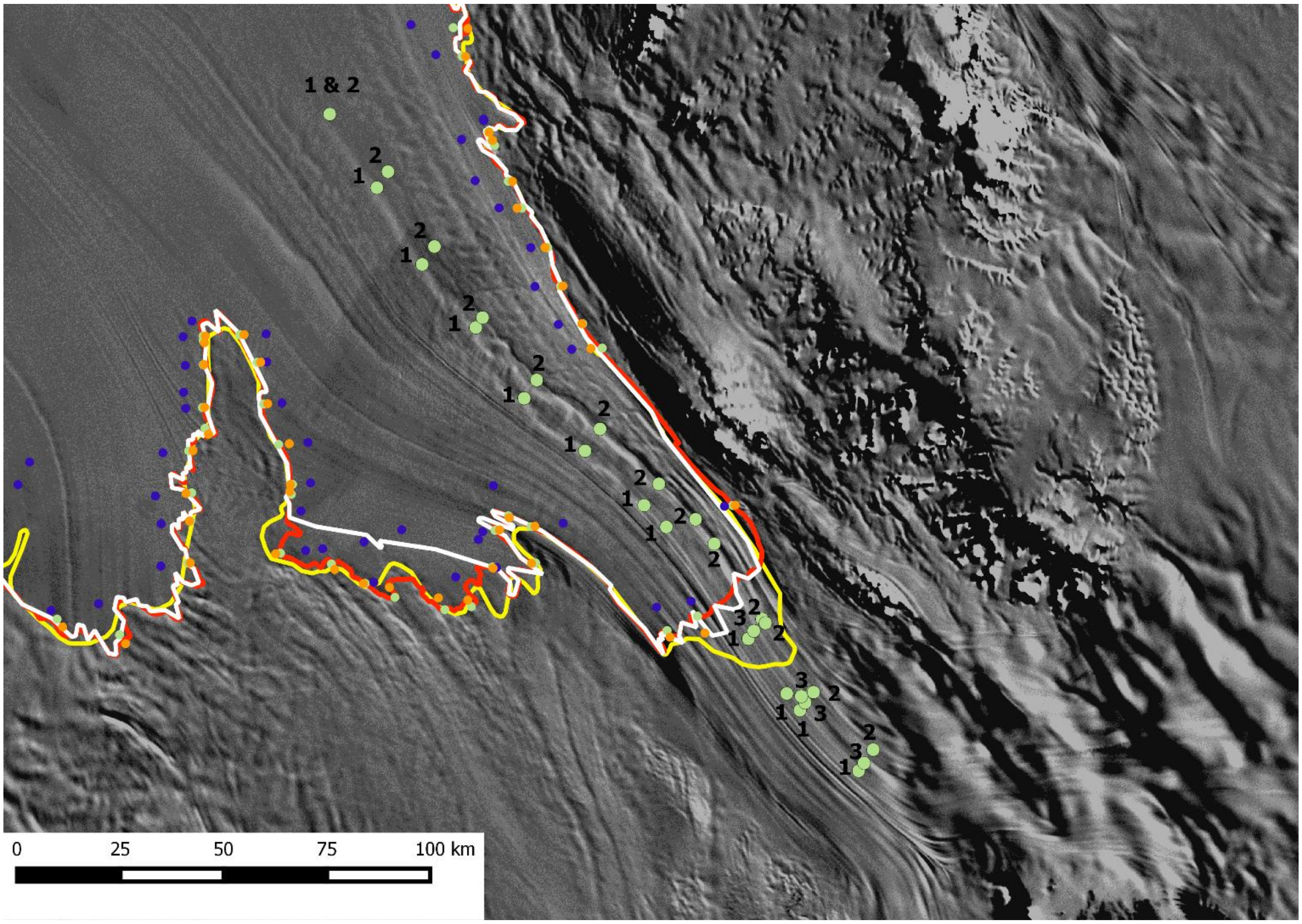

Supplementary Figure 6. The locations of subglacial channels labelled in Supplementary Figure 5 and in radar profiles in Figure 2 of the main paper. Grounding lines are as in Supplementary Figure 4.

Supplementary Note 2. Reflectivity over the 800 m peak

We examine the radar powers returned from two points of the ice sheet base, located in the radargram provided in Supplementary Figure 7.

At 1: AGL h1 = 648 m, power pw1 = -80 dB, depth z1 = 1581 m. At 2: AGL h2 = 681 m, power pw2 = -104.2 dB, depth z2 =2310 m. Power difference: pw2-pw1 = -24.2 dB; depth difference: z2-z1 = 729 m. Spreading loss difference:

G2-G1 = 10*log10{[h1+z1/sqrt(3.15)]/[h2+z2/sqrt(3.15)]}^2 = -2.2 dB (specular reflection)

G2-G1 = 10*log10{[h1+z1/sqrt(3.15)]/[h2+z2/sqrt(3.15)]}^4 = -4.4 dB (distributed scattering)

Ice attenuation loss difference: L2-L1 = -9.5*(z2-z1)*2/1000 = -13.9 dB

pw2-pw1 = (G2-G1) + (L2-L1) + (R2-R1)

So, the reflectivity difference: (R2)^2-(R1)^2 = (pw2-pw1) - (G2-G1) - (L2-L1) = -24.2+2.2+13.9 = -8.1 dB, this corresponds to a dielectric constant of 9.3 of the basal material at 2 assuming 1 is reflected from water.

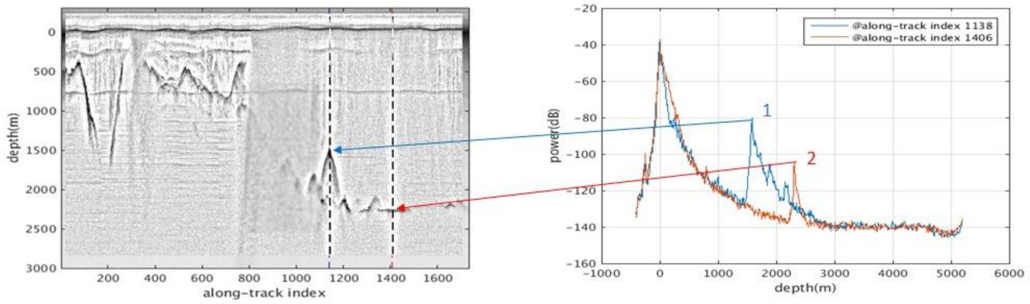

Supplementary Figure 7. Radargram and reflection powers for a section of line I-I' (Figure 2, main paper), in which the 800 m basal peak is observed in the grounding zone of Foundation Ice Stream.

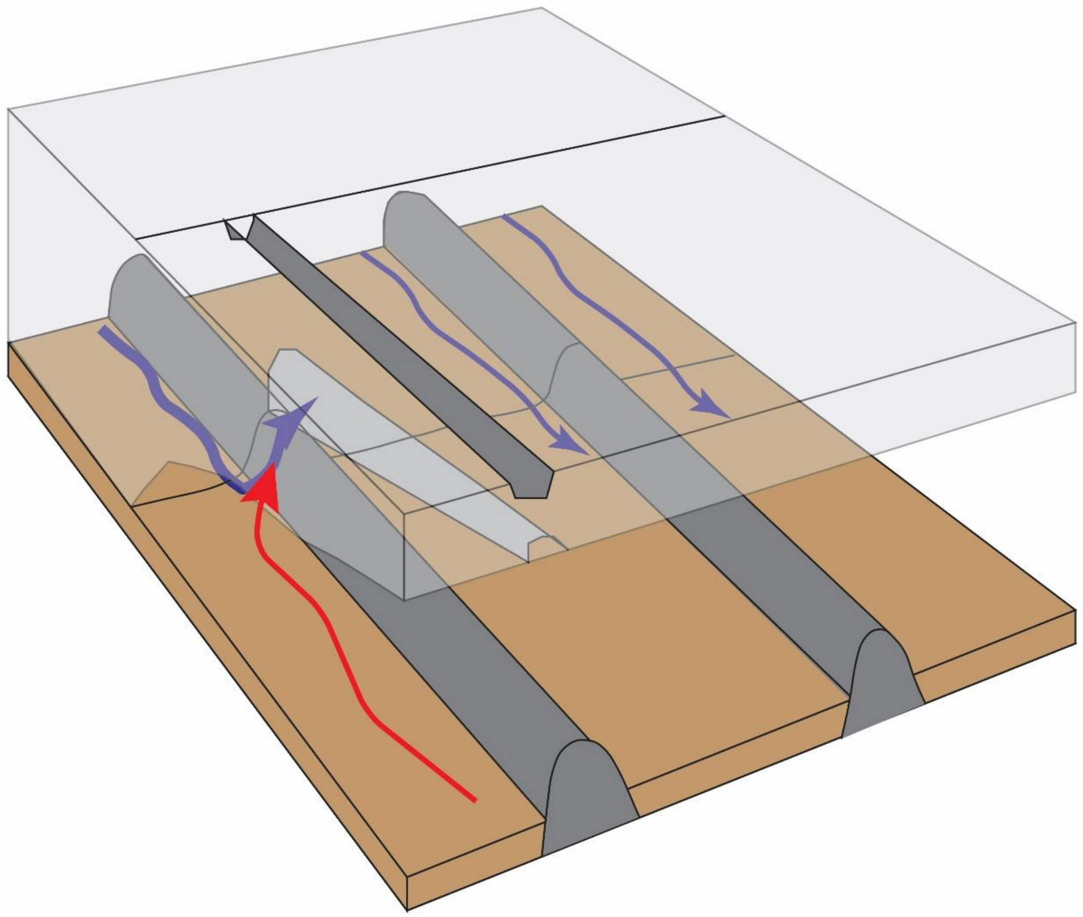

Supplementary Figure 8. Cartoon showing how flow-parallel subglacial landforms can corrugate the ice-shelf at the grounding line to form a U-channel, and dictate the position of subglacial water emerging from the grounded ice sheet. Cold basal water (blue arrow) mixes with dense warmer ice-shelf cavity water (red arrow) and rises upwards into the U-channel. The water then flows toward the margin, influenced by tidal flow of water, excavating the U-channel further.

Supplementary References

[1] Fretwell, P. et al. Bedmap2: improved ice bed, surface and thickness datasets for Antarctica. *The Cryosphere*. **7**, 375-393 doi:10.5194/tc-7-375-2013 (2013).

[2] Brunt, K.M., Fricker, H.A., Padman, L. & O'Neel, S. ICESat-Derived Grounding Zone for Antarctic Ice Shelves, Boulder, Colorado USA: National Snow and Ice Data Center, Digital media, doi:10.7265/N5CF9N19 (2010).

[3] Bindschadler, R. et al. High-resolution image-derived grounding and hydrostatic lines for the Antarctic Ice Sheet, Digital media, National Snow and Ice Data Center, Boulder, Colorado, USA, doi: dx.doi.org.10.7265/N56T0JK2 (2011).

[4] Rignot, E., Mouginot, J. & Scheuchl, B. MEaSUREs Antarctic Grounding Line from Differential Satellite Radar Interferometry, Version 2. National Snow and Ice Data Center: Boulder, CO, USA, doi:10.1029/2011GL047109. (2016).

[5] Bohlander, J., & Scambos, T. Antarctic coastlines and grounding line derived from MODIS Mosaic of Antarctica (MOA), National Snow and Ice Data Center, Boulder, CO, USA. (2007).

[6] Rodriguez-Morales, F. et al. Advanced multifrequency radar instrumentation for polar Research. *IEEE Trans. Geosci. Rem. Sens.* **52**, 2824–2842 (2014).

[7] Jordan, T.M., Cooper, M.A., Schroeder, D.M., Williams, C.N., Paden, J.D., Siegert, M.J. & Bamber, J.L. Self-affine subglacial roughness: consequences for radar scattering and basal water discrimination in northern Greenland. *The Cryosphere*. **11**, 1247-1264 doi:10.5194/tc-11-1247-2017 (2017).

[8] Oswald, G. & Gogineni, S. Recovery of subglacial water extent from Greenland radar survey data. *J. Glaciol.* **54**, 94–106 (2008).

[9] MacGregor, J.A., Winebrenner, D.P., Conway, H., Matsuoka, K., Mayewski, P. A. & Clow, G. D. Modeling englacial radar attenuation at Siple Dome, West Antarctica, using ice chemistry and temperature data. *J. Geophys. Res.* **112**, F03008 (2007).

[10] Matsuoka, K., MacGregor, J.A., & Pattyn, F. Predicting radar attenuation within the Antarctic ice sheet. *E. Plan. Sci. Lett.* **359-360**, 173–183 (2012a).

[11] Matsuoka, K., Pattyn, F., Callens, D. & Conway, H. Radar characterization of the basal interface across the grounding zone of an ice-rise promontory in East Antarctica. *Ann. Glaciol*. 53, 29–34 (2012b).
